# Navigation of a Telepresence Robot via Covert Visuospatial Attention and Real-Time fMRI

**DOI:** 10.1007/s10548-012-0252-z

**Published:** 2012-09-11

**Authors:** Patrik Andersson, Josien P. W. Pluim, Max A. Viergever, Nick F. Ramsey

**Affiliations:** 1 Image Sciences Institute, University Medical Center Utrecht, Utrecht, The Netherlands; 2 Division of Neuroscience, Department of Neurology and Neurosurgery, Rudolf Magnus Institute of Neuroscience, University Medical Center Utrecht, Utrecht, The Netherlands

**Keywords:** Brain--computer interface, Real-time fMRI, Visuospatial attention, Multivariate analysis

## Abstract

Brain–computer interfaces (BCIs) allow people with severe neurological impairment and without ability to control their muscles to regain some control over their environment. The BCI user performs a mental task to regulate brain activity, which is measured and translated into commands controlling some external device. We here show that healthy participants are capable of navigating a robot by covertly shifting their visuospatial attention. Covert Visuospatial Attention (COVISA) constitutes a very intuitive brain function for spatial navigation and does not depend on presented stimuli or on eye movements. Our robot is equipped with motors and a camera that sends visual feedback to the user who can navigate it from a remote location. We used an ultrahigh field MRI scanner (7 Tesla) to obtain fMRI signals that were decoded in real time using a support vector machine. Four healthy subjects with virtually no training succeeded in navigating the robot to at least three of four target locations. Our results thus show that with COVISA BCI, realtime robot navigation can be achieved. Since the magnitude of the fMRI signal has been shown to correlate well with the magnitude of spectral power changes in the gamma frequency band in signals measured by intracranial electrodes, the COVISA concept may in future translate to intracranial application in severely paralyzed people.

**Electronic supplementary material:**

The online version of this article (doi:10.1007/s10548-012-0252-z) contains supplementary material, which is available to authorized users.

## Introduction

The concept of Brain–Computer Interfaces (BCI) concerns technologies creating direct communication channels between the brain and a computer or other type of device. The goal is to accomplish real-time decoding of brain activity with sufficient reliability for paralyzed people to use it in their daily life. Two essential and defining components in a BCI system are the modality used for acquiring brain signals and the mental control tasks used for regulating this activity. With regards to signal acquisition, the main focus has so far been on electroencephalography (EEG). However, the implicit disadvantages of EEG, such as a low spatial resolution and high sensitivity to non-neural electrical activity, have led to a growing interest in intracranial acquisition techniques. By implanting intra-cranial electrodes, the quality, bandwidth and spatial resolution of the signal can be increased significantly.

The mental control tasks have mainly been based on brain functions that involve strong signals, such as the motor potential and the P300 oddball response (Wolpaw et al. 2002), because these can be detected well from the scalp using EEG. The improved signal quality of intracranial technologies allows testing brain functions not previously used for BCI (Leuthardt et al. 2009; Vansteensel et al. 2010; Gunduz et al. 2011). Some BCI users find currently employed brain functions hard or even impossible to control and some brain functions might be more intuitive for certain BCI applications. It is therefore necessary to evaluate new BCI control paradigms. Recent studies indicate the potential of a new approach using top–down regulation of the sensory cortices via attention (Gunduz et al. 2011; Andersson et al. 2011). Attention can change brain activity even in the absence of exogenous stimuli (Kastner et al. 1999; Heinemann et al. 2009). Attending to a region of the peripheral visual field, while keeping the gaze fixed, generates neural responses in the parts of the cortex processing visual information from this region (Brefczynski-Lewis et al. 2009; Datta and DeYoe 2009). We have previously shown that it is possible to decode, with high fidelity, individual fMRI images in real time during covert visuospatial attention (COVISA) (Andersson et al. 2011). It is important that the feasibility of a BCI control strategy is tested with a closed-loop system. In real life, BCI control will coincide with the processing of external stimuli. It is only when the test subject is exposed to this potentially interfering coincidence that the control strategy can truly be evaluated.

Covert visuospatial attention would constitute a very intuitive brain function for spatial navigation. In the present study we test the hypothesis that people can navigate a robot in realtime by merely shifting the visuospatial attention, without moving the eyes and without the need for exogenous stimuli. The subjects were instructed to navigate the robot through a course containing targets that were to be reached in a particular order. Our robot is equipped with a camera and the images are sent as feedback to the user. We used an ultrahigh field MRI scanner (7 Tesla) to obtain an fMRI BOLD (blood oxygen level dependent) signal that is strong enough for real-time decoding. Since BOLD activity is well correlated spatially with changes in the higher frequencies of electrophysiological signals (Lachaux et al. 2007; Hermes et al. 2012), the performance with fMRI is an indirect indication of the feasibility of a BCI with electrode implants.

## Materials and Methods

### Subjects

Four healthy volunteers (age 20–50, right-handed, 2 male) with normal or corrected-to-normal vision participated in the study, after giving their written informed consent. The study was approved by the ethics committee of the University Medical Center Utrecht in accordance with the declaration of Helsinki (2008). Each subject was scanned three times (one practice session and two performance sessions) separated by 1–28 days.

### Robot

We used the Erector Spykee robot (Meccano Toys Ltd) that is equipped with a wireless modem and a video camera. The software was designed such that a forward movement instruction moved the robot 50 cm forward, while a right or left instruction turned it 30^. There was a small variation in the size of the actual movements, given that the robot was designed for recreational use. The robot had no mechanism preventing it from hitting the wall. On a few occasions it moved forward and locked itself in place with the front against the wall. When that happened, it was moved back manually to the previous position and orientation.

### Data

The subjects were scanned at a 7T Philips Achieva system with a 16-channel headcoil, which generates the signal quality needed for our purpose (Andersson et al. 2011). The functional data were recorded using an EPI sequence (TR/TE = 1620/25 ms; FA = 90; SENSE factor = 2; 35 coronal slices, acquisition matrix 96 × 96, slice thickness 2 mm with no gap, 1.848 mm in-slice resolution). The field of view (FOV) was selected such that it covered the occipital lobe and the most posterior part of the parietal lobe. A high-resolution image was acquired for the anatomy using a T_1_ 3D TFE sequence (TR/TE = 6/2 ms; FA = 7; FOV = 220 × 180 × 200 mm; 0.55 × 0.55 × 0.5 mm reconstructed resolution).

### Experimental Setup

**Fig. 1:**
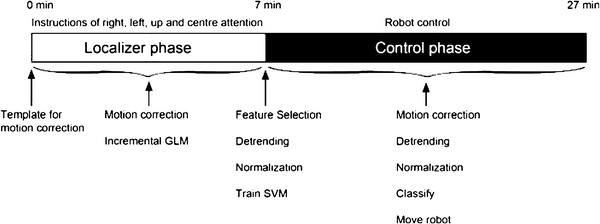
Illustration of the experiment timeline

Each session consisted of a single fMRI run of 995 image volumes. The first 270 volumes, the localizer phase, were used for locating relevant voxels and training the classifier. The remaining 725 volumes, the control phase, were classified as commands to control the robot. During the localizer phase the subjects were instructed where to covertly direct their visuospatial attention. Trials of right, left and up attention were randomized, and were always separated by a center attention trial. Each trial was 8.1s (5 TRs) long. During the control phase no instructions were given and the subject could move the attention at will. Directing the attention upward now made the robot move forward while directing the attention to the left and right resulted in a turn to the respective direction. The subjects were instructed to keep the gaze fixated at the center of the screen during the complete experiment. A timeline of the experiment, showing the different steps of the online analysis, can be found in Fig. 1.

### Task and Navigation Interface

**Fig. 2:**
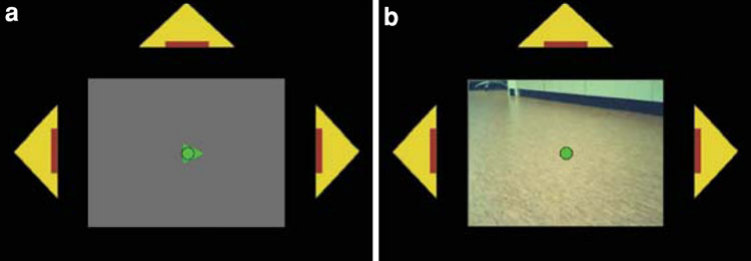
The feedback screen. The screen projected to the user during **a** the localizer phase, and **b** the control phase. The *three yellow triangles* served as targets for left, right and up attention. The *green circle* in the *center* indicates the point upon which the gaze had to be focused at all times. During the localizer phase the subjects direct their attention in response to a central cue (**a** shows the cue for right attention). During the control phase the video from the robot’s camera was displayed in the central area (Color figure online)

During the experiment the subjects were presented with an image as in Fig. 2, projected onto a small projection screen in the bore of the scanner. Subjects were instructed to, at all times, fixate their gaze on a circle displayed at the center of the screen. Three yellow triangles permanently positioned to the two sides and to the top of the central area indicated the attention target areas, used for sending commands. During the localizer phase (Fig. 2a) the center circle alternately changed into a cue (an arrow) pointing towards one of the yellow target areas, and then back to a circle. Subjects were instructed to covertly direct their attention to the target area located in the direction of the instruction arrow or, in the case of a circle, to focus their attention on the center. During the control phase the live video images were displayed in the area between the attention targets (Fig. 2b) and there was no cue to indicate where to direct the attention to. The BOLD signal exhibits a slow response to neuronal activity, and it takes several seconds after an instruction has been identified for the signal to return to baseline (Andersson et al. 2011, 2012; Siero et al. 2011). The attention therefore needed to return to the center immediately after the execution of a robot movement to let the hemodynamic effect wash out before the next command could be sent. To facilitate this, the video was turned off during four volumes (6.48 s) after a volume had been classified as either right, left or up attention and the corresponding movement had been executed. Pilot tests revealed that, although the hemodynamic response takes longer than that to completely disappear, the BOLD signal has stabilized enough for a new command to be sent.

### BCI Hardware

**Fig. 3:**
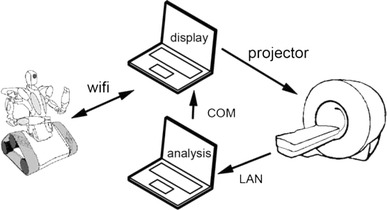
The BCI system. The system consists of the MR scanner, two computers and the robot

The BCI system consisted of two computers communicating in real time with each other, with the MR scanner and with the robot (see Fig. 3). One computer received the images from the scanner directly after reconstruction via the local network using a TCP/IP protocol and the Philips DRIN (Direct Reconstruction INterface) module. This computer performed the main analysis (motion correction, detrending, SVM training, classification etc). The second computer controlled the graphic display, projected to the subject via a video projector. The display was updated according to instructions from the first computer via a serial cable. The second computer also contained the wireless link to the robot for communicating the video images and the movement commands. The graphic display and robot communication were implemented using the RoboRealm software (http://www.roborealm.com).

### Motion Correction

All image volumes were corrected for head movements. Motion during the localizer phase will result in a weaker classifier, and during the control phase the wrong features will be extracted from the image volume and sent as input to the classifier. Every volume was rigidly registered to the first localizer volume. Before the fitting, the images were smoothed with a Gaussian filter (σ = 1 voxel). As similarity metric we used the sum of squared differences. The optimization scheme consisted of 50 iterations of the stochastic gradient descent method described in Klein et al. (2007). The final image was generated using cubic B-spline interpolation. The code was implemented in C++ and was compiled to a Matlab (Mathworks, Natick, MA) mex-file.

### Feature Selection

Each sample of fMRI data, i.e. each volume, contains a very large number of voxels of which the majority are either located outside the brain or are not involved in processing the attention task. In order to avoid overfitting the classifier model, a feature selection step is necessary before it is built. Overfitting occurs when the classifier is trained on voxels that contribute with information that is irrelevant for determining the attention state. Our voxel selection is based on a GLM analysis that runs throughout the localizing part. Four statistical t maps were incrementally updated with every new image using the algorithm described in Bagarinao et al. (2003). The GLM model contained five regressors; right, left, up and center attention, plus a linear drift term. The t values were computed using the contrasts ‘one minus the others’. That is, for right attention the contrast was ‘$\hbox{right} - \frac{1}{3}$ [left + up + center]’ etc. After the last iteration of updating the t maps, a voxel selection was performed in two steps. A first selection was made by merging the voxels with the 500 highest values from each of the four t maps. Second, from this first selection clusters smaller than 5 voxels were removed. The remaining pool of voxels was subsequently available for the SVM to train on.

It is possible that the use of a multivariate method such as Recursive Feature Elimination (De Martino et al. 2008), using the actual classification model, to select voxels could result in a slightly better performance. However, the computation would take much longer and we would not be able to combine both the localizer and control part in a single fMRI run.

### SVM Classifier

We used the LIBSVM (Chang and Lin 2011) implementation of a C-SVM classifier with a linear kernel and the regularization parameter *C* = 1. Theoretically, if *C* is too large, we risk overfitting, and if it is too small, underfitting. However, it has been shown that the classification result is rather insensitive to the value of *C* (LaConte et al. 2005), and the unit value is often used. LIBSVM uses the "one-against-one" approach for multiclass problems. This means that our classifier consisted of six binary SVMs, one for each pair of classes (attention directions), and an image was assigned to the class with the majority vote. In case of a tie we classified it as center attention (thus no action was taken by the robot).

### Signal Detrending and Normalization

fMRI signals always contain low-frequency drift to various degrees. To minimize the influence of these signal changes on the classification we applied detrending to the data. For this we used an implementation of the algorithm described in Tarvainen et al. (2002) with regularization parameter λ = 200. As soon as the last image volume of the localizer phase had been analyzed and the feature selection was ready, the complete time series of the selected voxels were detrended. From the detrended data we then estimated the baseline and standard deviation for each voxel. Using these estimates the amplitude of each voxel’s time series was normalized to have zero mean and unit variance. The detrended and normalized signals were then finally used for training the SVM. The original non-detrended values were kept in memory so that they could be used in the detrending of later image volumes. During the control phase, as soon as a new volume had been passed on from the MRI scanner and had been registered to the template image, the signal values were detrended and normalized in the same way as during the training. The processed values were then classified by the SVM.

### Practice Session

The purpose of the practice session was to acquaint the subjects with the robot control environment and the slow response inherent to fMRI based BCI. Each subject was asked to try different lengths of attention to find out what produced the best results.

### Evaluation Sessions

**Fig. 4:**
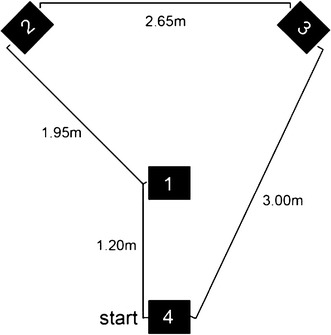
Map of the robot control environment with the positions of the four targets. The robot started at target four and the instructions were to reach the targets in sequence

In the two evaluation sessions the robot was placed in the same room as in the practice session. Four targets (25 × 50 cm) were distributed in the room as seen in Fig. 4, marked out on the floor and labeled with the numbers 1–4. The instructions were to move the robot to these targets in sequence, and to continue until the time was up once target four was reached. The time of each target reached was recorded as one of the measures of performance.

## Results

### Feature Selection

In the feature selection we merged 500 voxels from each of the four t maps. However, due to partial overlaps and the removal of clusters with less than five voxels, the final selection consisted of fewer than 2,000 voxels. Table 1 shows the number of voxels selected and used in the SVM training in each of the sessions. The average number of voxels included was 1,236, which corresponds to a volume of 8.4 cm^3^.

**Fig. 5:**
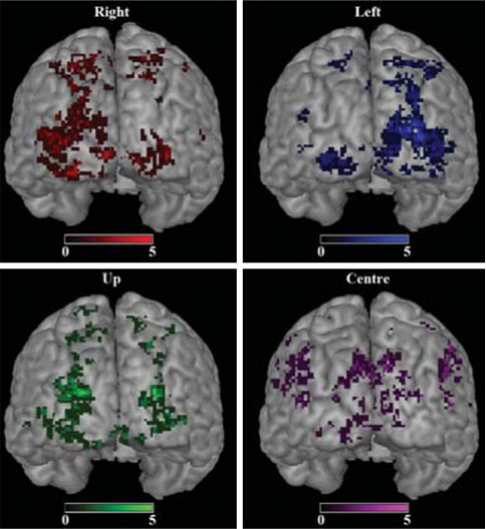
Voxels selected in the online GLM analysis, displayed on the Montreal Neurological Institute (MNI) reference brain. Four statistical t maps, each corresponding to an attention direction, were computed online during data acquisition. From each of these t maps a mask was created by first locating the 500 highest t values and then removing any cluster smaller than five voxels. In the online analysis the masks were merged to create the voxel selection to train the SVM on. In this figure, the masks from all subjects were spatially normalized and added, separately for each attention direction. It should be noted that the spatial normalization was applied only for illustration purposes and was not a part of the online analysis. With four subjects and two (performance) sessions each, the sum could take values between 1 and 8. However, since no voxel was selected in more than five sessions, the scale of the overlays is adjusted to this value

Figure 5 shows group maps of the voxels selected from each attention direction (before they were merged to a single selection). The group maps were created by first spatially normalizing each subject’s anatomical image to the Montreal Neurological Institute (MNI) reference space and then applying the computed transformation to the mask defining the selected voxels. Finally, all subjects’ normalized masks were added up to display how often a certain voxel was selected.

**Table 1:** The number of features selected by the GLM feature selection during the practice session (P) and the two evaluation sessions

Subject$\setminus$session	P	1	2
1	1,404	1,313	1,501
2	1,265	1,132	1,214
3	1,214	1,076	988
4	986	1,437	1,298

## Performance

Table 2 shows the performances of all subjects in both of the evaluation sessions. The performance was measured by the number of targets reached and the number of movements and time required to reach them. All subjects managed to reach at least three of the four targets, and the maximum number of targets reached was six. With 725 images and a TR of 1.62 s, the complete control phase lasted nearly 20 min (1,175 s). If we assume that the minimum time between two commands is 10 TRs (16.2 s, including five TRs for the BOLD signal to reach a detectable level, one TR for the movement and four TRs for the signal to return), the maximum number of commands that can be sent during the experiment is 72. The two best subjects, reaching five and six targets, did so using 71 and 67 movements respectively.

**Fig. 6:**
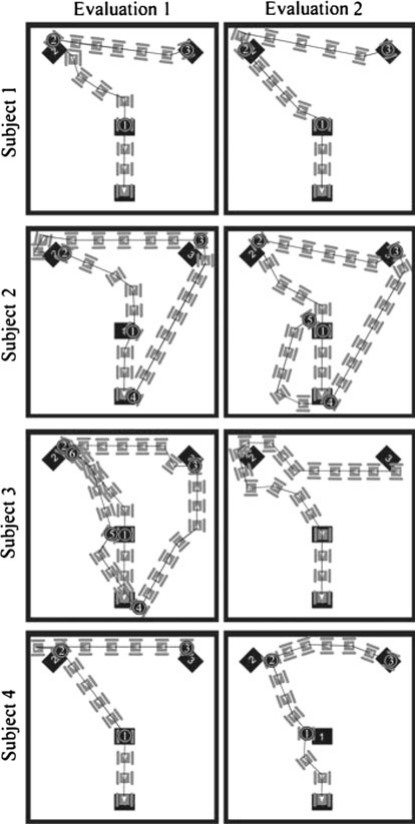
The robot’s paths during the navigation. The path is only shown to the point where the last target was reached. Only the forward movements are shown, and a sequence of instructions such as right-left-forward is displayed as one forward movement. Each *gray* "robot symbol" symbolizes the position after a forward movement. The robot’s position when hitting a target is indicated with a circle containing the target number

Figure 6 visualizes how the robot was maneuvered during the two sessions. Note that only the forward movements result in a new position. For example, a right turn followed by a left turn cancel each other out and is not visible in these maps. A video recording of one session (not part of the study) can be found in Supplementary Materials. In the video the robot moves five times the actual speed.

**Table 2:** The cumulative time (in seconds) from the start of the control phase until the targets were reached

Subject	Session	Target					
		1	2	3	4	5	6
1	1	81 (5)	407 (26)	899 (59)	–	–	–
1	2	109 (7)	287 (19)	689 (49)	–	–	–
2	1	109 (6)	298 (18)	748 (42)	1,042 (58)	–	–
2	2	70 (5)	196 (13)	483 (30)	833 (52)	1,123 (71)	–
3	1	47 (3)	243 (16)	570 (36)	818 (53)	936 (61)	1,034 (67)
3	2	87 (7)	279 (21)	716 (50)	–	–	–
4	1	32 (3)	120 (9)	927 (63)	–	–	–
4	2	209 (15)	510 (37)	1,011 (61)	–	–	–

The cumulative number of movements used for reaching the targets is shown within brackets

## Discussion

We have for the first time demonstrated real-time BCI control based on pure covert visuospatial attention, completely independent of eye movements and evoked responses. In a telepresence application, where a robot was navigated through a course containing four targets, the user communicated the intended movement by covertly directing the attention between four different regions in the visual field. Our four subjects were all able to control the robot and they reached at least three of the four targets. All subjects expressed the feeling of having control over the robot, even during the initial practice session. This supports the notion that COVISA based BCI control is intuitive and requires virtually no training (van Gerven and Jensen 2009; Andersson et al. 2011; Treder et al. 2011a). Although our study is the first demonstration of an applied BCI based on the visual system that is completely free from evoked responses and does not require eye movements, the concept of employing the visual system is not new. One example is BCI based on the steady state visually evoked potential (SSVEP). SSVEP is an evoked response present during a flickering stimulation of the retina, and is detected via an increase of power in the EEG or MEG signal at the frequency of the stimuli. The P300 is another event related potential (ERP) that has been used for BCI. This response occurs approximately 300 ms post-stimulus upon rare events. The matrix speller first described by Farwell et al. (Farwell and Donchin 1988), is a BCI based on the P300 visual response in EEG signals. Besides being intrinsically dependent on external visual stimulation, there is growing evidence that visual P300 and SSVEP BCI systems are more or less dependent on gaze control, yielding better results if subjects direct their gaze to the target as opposed to fixating gaze elsewhere (Allison et al. 2008; Shishkin et al. 2009; Bianchi et al. 2010; Brunner et al. 2010; Treder and Blankertz 2010).

For safety reasons inherent to the high magnetic field, we could not bring an eye tracker into the scanner environment (Andersson et al. 2011). Thus, we could not get online measures of eye movements. However, it has been shown quite often that people have no trouble performing covert spatial attention shifts without any eye movements (Brefczynski and DeYoe 1999; Siman-Tov et al. 2007; Munneke et al. 2008; Datta and DeYoe 2009; van Gerven et al. 2009; Andersson et al. 2011). Moreover, the brain activity patterns obtained during BCI strongly suggest (Andersson et al. 2011) that the subjects controlled the robot via covert shifting of attention, and not with eye movements. It is well known that covert shifting of attention to one side induces elevated activity in the contralateral visual cortex (Brefczynski and DeYoe 1999; Brefczynski-Lewis et al. 2009; Perry and Zeki 2000). As can be seen in Fig. 5, the bulk of activity is contralateral for left and right attentional shifts. If eye movements were used to control the robot, we would expect opposite results, since most of the visual information would shift to the hemifield opposite to the direction of eye movement, causing activity in the visual cortex ipsilateral to that direction. Up and down shifting is associated with inferior and superior visual cortex activation, respectively. Again the activity patterns are in agreement. To classify each image volume we trained a support vector machine on the initial localizer data. The application of multivariate classification techniques on fMRI data has been shown effective in multiple studies, e.g. (LaConte et al. 2005, 2007; Sitaram et al. 2011). Since fMRI volumes usually include a very large number of voxels, a feature selection step is most often included to remove uninformative voxels and avoid overfitting. Our feature selection was based on an online univariate GLM analysis. A multivariate feature selection method could potentially create a map more optimized for the SVM classifier, but our strategy is fast, and it allowed us to finish the feature selection and training within a single TR. The overlap of selected voxels across sessions shows that some regions in expected parts of the cortex are consistently selected (Fig. 5). Around these "hot-spots" there are voxels selected in only a few sessions. There can be several reasons for this distribution. First, visual field maps vary considerably across individuals (Dougherty et al. 2003; Yamamoto et al. 2012). Second, alignment of the functional data from the two different sessions and during the spatial normalization may not have been perfect, causing an apparent shift. Third, there could be small variations in where in the visual fields subjects directed their attention. They reported that they tried different strategies in order to feel confident in directing their attention. These strategies included imagining a beam of light shining from the center onto the target of interest, and pretending to expect a symbol to show up at the target. A change of strategy could potentially result in variations of selected voxels. It is also possible that the brain activation pattern changes in the course of learning to control the BCI. The current study with only three sessions does not allow an adequate assessment of this effect. We are planning a study with multiple sessions aimed at elucidating this particular topic.

Several BCI systems built on fMRI have been described (Yoo et al. 2004; Sitaram et al. 2007; Moench et al. 2008; Sitaram et al. 2008; Sorger et al. in press). These systems can for instance, as in this study, be employed for evaluating new BCI control paradigms or for determining the best choice of brain function for a specific patient population. However, the ultimate goal is to develop a BCI system that can function in every-day life for patients. Clearly MRI is then no longer an option, so implementation in a portable system is required to bring the technology to paralyzed users. Given the detailed distribution of activated brain areas it is unlikely that our results could be repeated using scalp electrodes. Instead, intracranial recordings may prove to be effective (Andersson et al. 2011). For successful BCI control, the responses to each of the attention directions need to be distinguished reliably, not only from each other but also from visual input provided by the video feedback. As seen in the activation maps, and as predicted by retinotopic studies, multiple cortical regions corresponding to the multiple visual maps become active during each direction of attention. The brain response to the central input provided by the video camera is strong and spatially close to the attention modulated effects. Thus, the implicit limitations in terms of resolution and signal strength will probably make EEG ineffective.

Both EEG and MEG have been used for investigating covert visuospatial attention for BCI control (Kelly et al. 2005; van Gerven and Jensen 2009; Treder et al. 2011a). However, none of these studies demonstrated real-time online decoding or visual feedback of the performance. It should also be noted that since MEG systems are not portable, BCI systems built on this technology can not be used in the every-day life of patients (similar to fMRI based systems). In a recent study Treder et al. (2011b) used EEG to implement a (ERP dependent) BCI speller based on both spatial and feature (color) attention, not dependent on eye movements. They evaluated two variants of speller interfaces that were sensitive to spatial attention and one that was not. They found the best performance in the version that was not sensitive to spatial attention. For the other two variants, incorporating both spatial and feature attention, the performance dropped substantially when only using the occipital electrodes. This suggests that they did not succeed in detecting the brain response to spatial attention.

Functional near-infrared spectroscopy (fNIRS) is an optical technique that measures the localized oxygenation level in the cortex via light emitters and sensors placed on the scalp. fNIRS systems are portable and can therefore be used for BCI (see review in Matthews et al. 2008) at home in patients’ daily life. However, this technique measures at a much lower spatial resolution than fMRI and is limited to the cortical regions close to the scalp. Thus, for the same reasons as for EEG it will be hard to separate attention towards multiple directions using fNIRS.

Intracranial recordings would most likely be suitable for COVISA BCI. These techniques can provide both high spatial resolution and give access to the higher frequencies that are too weak to be detected using scalp electrodes. The power in the gamma band (65–95 Hz) has been shown to correlate well with the BOLD signal (Lachaux et al. 2007; Hermes et al. 2012). Real-time fMRI can therefore facilitate BCI training and the activation pattern is likely to indicate the most reliable implant sites (Vansteensel et al. 2010) and make it possible to limit the cortical area that needs to be covered with electrodes for decoding. In the present study the attention was maintained directed at the targets for several seconds, allowing for the BOLD effect to build up. This would no longer be necessary when classifying electrophysiological signals. Hence, for an intracranial BCI system short shifts of attention may well be sufficient.

In a recent study (Gunduz et al. 2011) covert visual attention was studied with electrocorticography (ECoG) using a classical cueing task. Distinct foci of activity were found indicating that the associated brain signals were readily detectable. Moreover, in a previous paper (Andersson et al. 2011) we obtained a performance of 70 % with post-hoc offline analysis of ECoG data recorded during a two-direction visual attention task.

A benefit of COVISA based BCI control is that more directions can be added to achieve a more detailed BCI control, as long as the responses can be separated. Moreover, the concept allows for optimizing the brain signals (and discrimination thereof) by adjusting the positions of the attention target regions in the visual field. In conclusion, we have shown that navigation of a robot in realtime is feasible with COVISA BCI. Given that the center video display did not interfere with the generation of movement instructions for the robot, covert shifting of attention to the periphery can be performed without interfering with processing of information in the center of the field. Conceptually, more than the current three directions can be decoded (diagonal directions or even more), but this requires further investigation.

## Electronic supplementary material

Below is the link to the electronic supplementary material.

Supplementary material 1 (AVI 1,679 kb). The image in the lower left corner shows what the user is seeing. In the video the robot moves five times normal speed
